# US Obstetrician-Gynecologists' Perceived Impacts of Post–*Dobbs v Jackson* State Abortion Bans

**DOI:** 10.1001/jamanetworkopen.2023.52109

**Published:** 2024-01-17

**Authors:** Erika L. Sabbath, Samantha M. McKetchnie, Kavita S. Arora, Mara Buchbinder

**Affiliations:** 1 School of Social Work, Boston College, Chestnut Hill, Massachusetts; 2 Center for Work, Health, and Wellbeing, Harvard T. H. Chan School of Public Health, Boston, Massachusetts; 3 Department of Obstetrics and Gynecology, University of North Carolina at Chapel Hill School of Medicine, Chapel Hill; 4 Center for Bioethics, Department of Social Medicine, University of North Carolina at Chapel Hill School of Medicine, Chapel Hill

## Abstract

**Importance:**

Following the *Dobbs v Jackson Women’s Health Organization* decision in June 2022, 17 US states have functionally banned abortion except in narrow circumstances, and physicians found in violation of these laws face felony charges, loss of their medical license, fines, and prison sentences. Patient impacts are being studied closely, but less research has focused on the consequences for obstetrician-gynecologists (OB-GYNs), for whom medically necessary care provision may now carry serious personal and professional consequences.

**Objective:**

To characterize perceptions of the impact of abortion restrictions on clinical practice, moral distress, mental health, and turnover intention among US OB-GYNs practicing in states with functional bans on abortion.

**Design, Setting, and Participants:**

This qualitative study included semistructured, remote interviews with OB-GYNs from 13 US states with abortion bans. Volunteer sample of 54 OB-GYNs practicing in states that had banned abortion as of March 2023.

**Exposure:**

State abortion bans enacted between June 2022 and March 2023.

**Main Outcomes and Measures:**

OB-GYNs’ perceptions of clinical and personal impacts of abortion bans.

**Results:**

This study included 54 OB-GYNs (mean [SD] age, 42 [7] years; 44 [81%] female participants; 3 [6%] non-Hispanic Black or African American participants; 45 [83%] White participants) who practiced in general obstetrics and gynecology (39 [72%]), maternal-fetal medicine (7 [13%]), and complex family planning (8 [15%]). Two major domains were identified in which the laws affected OB-GYNs: (1) clinical impacts (eg, delays in care until patients became more sick or legal sign-off on a medical exception to the ban was obtained; restrictions on counseling patients on pregnancy options; inability to provide appropriate care oneself or make referrals for such care); and (2) personal impacts (eg, moral distress; fears and perceived consequences of law violation; intention to leave the state; symptoms of depression and anxiety).

**Conclusions and relevance:**

In this qualitative study of OB-GYNs practicing under abortion bans, participants reported deep and pervasive impacts of state laws, with implications for workforce sustainability, physician health, and patient outcomes. In the context of public policies that restrict physicians’ clinical autonomy, organization-level supports for physicians are essential to maintain workforce sustainability, clinician health and well-being, and availability of timely and accessible health care throughout the US.

## Key Points

**Question:**

How do obstretrician-gynecologists (OB-GYNs) perceive the impact of post–*Dobbs v Jackson* state abortion bans in affected states?

**Findings:**

In this qualitative study describing the experiences of 54 OB-GYNs practicing under abortion bans in 13 states, OB-GYNs described a range of perceived impacts, including distress at having to delay essential patient care, fears of legal ramifications, mental health effects, and planned or actual attrition.

**Meaning:**

These findings suggest that state abortion bans have impacted OB-GYNs, with implications for both patient outcomes and the sustainability of the OB-GYNs workforce.

## Teaser

This qualitative study examines the perceptions of obstetrician-gynecologists regarding the impact of abortion restrictions on clinical practice, moral distress, and mental health in the US.

## Introduction

In June 2022, the US Supreme Court’s *Dobbs v Jackson Women’s Health Organization* (*Dobbs v Jackson*) decision overturned the federal protection for abortion rights enshrined in *Roe v Wade.* As of December 2023, 16 US states have enacted functional bans on abortion, with limited exceptions for maternal health, rape, incest, or fatal fetal anomalies.^1,2^ Similar legislation is pending in 5 states.^3^

These restrictions have had profound public health consequences, affecting not only induced abortion rates, but also care for obstetric emergencies, miscarriage management, and treatment of serious illness during pregnancy.^4,5,6^ Early evidence suggest that abortion bans have lowered the standard of care and worsened patient health,^7^ amplifying socioeconomic, racial, ethnic, and geographic disparities in pregnancy outcomes.^8,9,10^

State abortion bans also have implications for clinicians engaged in reproductive health care, particularly obstetrician-gynecologists (OB-GYNs), who provide the bulk of US reproductive care.^11^ In the most restrictive states, illegally terminating a pregnancy is a felony, with consequences including loss of medical license and prison sentences.^12^ The ambiguous language of many laws within an untested legal environment may contribute to perceptions of legal risks^9^ and subsequent workplace stress. For example, physicians in states with aiding and abetting clauses are sometimes instructed that discussing abortion could lead to civil or criminal penalties.^13,14^

Prior to *Dobbs v Jackson*, many studies documented how state-level abortion restrictions imposed stressful burdens on clinicians who provide abortion care, amplifying abortion stigma and interfering with patient-clinician rapport, thereby contributing to occupational stress, compassion fatigue, and burnout.^15,16,17,18^ However, the broad nature of post–*Dobbs v Jackson* abortion bans have potential implications for all OB-GYNs in affected states, not just the 14% who provide abortion care.^19^ Commentaries have discussed potential impacts of abortion bans on OB-GYNs, pointing to likely increased documentation burdens, ethical challenges, and heightened stress when treating cases in legal gray areas.^11,20,21,22,23,24^ Workforce-related impacts also include retention and recruitment difficulties.^25,26,27,28^ A May 2023 survey found that 55% of Idaho OB-GYNs were seriously or somewhat considering leaving the state due to the abortion ban.^29^ However, there has been little systematic investigation of impacts of state abortion bans on OB-GYNs. The purpose of this study was to characterize the experiences of OB-GYNs practicing under state abortion bans, which is an underexplored dimension of the public health impacts of *Dobbs v Jackson*.

## Methods

This qualitative study, the Study of OB-GYNs in Post-*Roe* America (SOPRA) followed the Standards for Reporting Qualitative Research (SRQR) reporting guideline.^30^ SOPRA was approved by the institutional review board of Boston College. We received a waiver of documented informed consent and obtained verbal consent from all participants.

### Research Design

Participants were eligible if they practiced obstetrics and gynecology in any of the 14 states in which abortion became and remained illegal with limited exceptions from June 2022 to March 2023: Alabama, Arkansas, Georgia, Idaho, Kentucky, Louisiana, Mississippi, Missouri, Oklahoma, South Dakota, Tennessee, Texas, West Virginia, and Wisconsin. We included general OB-GYNs and subspecialists in maternal-fetal medicine and complex family planning. Trainees (medical students, residents, and fellows) were ineligible. Those who had worked in eligible states post–*Dobbs v Jackson* but had since relocated were classified according to their prior state.

### Recruitment

Prospective participants were identified and recruited from March to August 2023 using announcements on OB-GYN listservs and social media groups, professional networks, direct email recruitment, and snowball sampling. Prospective participants were purposively sampled to achieve balance across states, practice characteristics, and sociodemographic characteristics. Sample size was determined by a saturation of themes.

### Data Collection

We developed and piloted a semistructured interview guide covering 5 domains: (1) professional background; (2) perspectives on abortion bans; (3) impact of bans on clinical practice and experiences of moral distress; (4) personal health and well-being impacts; and (5) institutional practices and policies. We included questions about leadership experiences when relevant. Interviews were conducted by E.L.S (an epidemiologist) and M.B. (a medical anthropologist) via Zoom and lasted 60 to 75 minutes. Audio recordings were professionally transcribed and deidentified. Recordings were supplemented with fieldnotes with investigators’ reflections on key points raised in each interview. Participants received a $75 digital gift card. Prior to recording, participants completed a verbal questionnaire, self-identifying their gender, race, ethnicity, age, and professional characteristics. Responses were used to assess heterogeneity of experiences.

### Data Analysis

Transcripts were coded using a thematic qualitative approach,^31^ in which we assigned codes to blocks of text corresponding to code definitions using Dedoose version 9.0.107 (Dedoose).^32^ We used emergent themes from our fieldnotes to develop a structured coding dictionary, refining it iteratively during an initial training period in which 8 transcripts were coded by multiple coders to establish agreement on coding applications. The remaining 46 transcripts were each coded by 1 coder, with 1 of every 6 transcripts (8 transcripts [17%]) either double-coded or reviewed by a second coder. Uncertainties were discussed with the team to reach consensus. Coding and analyses were conducted from June to October 2023.

## Results

This study included 54 OB-GYNs (mean [SD] age, 42 [7] years; 44 [81%] female participants; 3 [6%] non-Hispanic Black or African American participants; 45 [83%] White participants) who practiced in general obstetrics and gynecology (39 [72%]), maternal-fetal medicine (7 [13%]), and complex family planning subspecialists (8 [15%]) (Table 1). Nine clinicians (17%) were in private practice, 40 (74%) were employed by a hospital (including academic), and 3 (5%) worked in federally qualified health centers (Table 2).

**Table 1.** Participant Social and Demographic Characteristics (N = 54)

Characteristics	Participants, No. (%)
Age, mean (SD) [range], y	42 (7) [33-66]
Gender	
Woman	44 (81)
Man	10 (19)
Nonbinary or other	0
Race and ethnicity	
American Indian or Alaskan Native	0
Asian	4 (7)
Non-Hispanic Black or African American	3 (6)
Hispanic or Latino	0
Non-Hispanic White	45 (83)
More than 1 race	1 (2)
Another race not specified	1 (2)
Subspecialty	
Generalist	39 (72)
Maternal-fetal medicine	7 (13)
Complex family planning	8 (15)
Practice type	
Employed by a hospital	40 (74)
Private practice	9 (17)
Federally qualified health center	3 (5)
Other	2 (4)
Years in practice, mean (SD) [range]	11 (7) [1-36]
Clinical leadership role	
Yes	8 (15)
No	46 (85)

**Table 2.** Participants’ Geographic and Institutional Characteristics (N = 54)

States	Participants, No. (%)
Alabama	4 (7)
Arkansas	1 (2)
Georgia	4 (7)
Idaho	8 (15)
Kentucky	1 (2)
Louisiana	2 (4)
Mississippi	0
Missouri	2 (4)
Oklahoma	6 (11)
South Dakota	5 (9)
Tennessee	7 (13)
Texas	7 (13)
Wisconsin	3 (6)
West Virginia	4 (7)
Institution type	
Academic medical center	28 (52)
Community hospital	26 (48)
Public institution	9 (17)
Catholic institution	6 (11)
Geographic setting	
Urban	41 (76)
Suburban	5 (9)
Rural	8 (15)
Proportion of OB patients covered by Medicaid, %	
0-25	6 (11)
26-50	20 (37)
51-75	19 (35)
76 or more	9 (17)
No. of beds in primary hospital	
Less than 250	10 (19)
250-500	14 (26)
More than 500	30 (56)

Abbreviation: OB, obstetrics.

### Clinical Impacts

#### Delayed Care

Many participants described needing to delay medically necessary care until patients were at risk of death or permanent impairment, or the fetal heart stopped spontaneously. One said, “The way our legal teams interpreted it, until they became septic or started hemorrhaging, we couldn’t proceed…[it] puts women in a very challenging, risky position. Is a 5% risk of death enough? Does it take 20%? Does it take 50%? What is enough legally? And the legal people seem to have a different definition that also just feels horrible, to say until you’re at a greater than likely chance of dying, you can’t make a decision.”

Clinically inappropriate delays, and their consequences, also occurred during middle-of-the-night emergencies when calls to legal teams for clearance to proceed went unanswered. One participant said, “You have somebody hemorrhaging with an intrauterine pregnancy with a heartbeat…I [didn’t yet] have legal coverage for that, but there’s only so many times you can transfuse somebody and they’re begging for their life before you say, ‘This is unconscionable.’”

Participants described the consequences of delays for both patients and themselves: “[The patient] wasn’t bleeding heavily enough for me to intervene… But she wasn’t stable [enough] to be transferred because she was actively laboring…I had to go into this woman’s room and be like, ‘I’m sorry, there’s nothing I can do for you other than make you comfortable. Hopefully, this will happen quickly for you.’ And we watched her labor for 12 hours and she did finally deliver, but there was nothing I could do.”

#### Counseling Restrictions

Due to aiding and abetting clauses, some participants were informed by their institution that they could not provide referrals for abortions or discuss abortion as an option. Many described such limits on counseling as the laws’ most damaging parts, calling these restrictions an affront to their medical expertise and to patient autonomy. Although most reported that they will counsel but not document discussions, such conversations felt risky: “[Hospital] even warned us, if you feel like you have a patient who may be pro-choice…you shouldn’t contribute to that conversation because you don’t know if you’re being recorded…they could use that against you.” They described scripts to counsel patients in ways that felt legally defensible: “In [STATE]…I can’t counsel you on termination, but I need you to understand that there are locations outside of [STATE] that don’t have the same laws.”

#### Inability to Provide Care

In the absence of counseling restrictions, participants could accurately describe care options, but these options were functionally unavailable to many patients: “[It’s] incredibly frustrating because it’s care and services that I am capable of providing to people and that are not unsafe or hard.” This was especially true for participants with a high proportion of low-income, Medicaid-insured patients who could not afford to travel out of state; a clinician said, “It just doesn’t feel very fair to patients…I have reviewed all options that are available, but then my hands are kind of tied in terms of them saying, ‘Well, how do I get this?’ Or, ‘What’s next?’ I feel I’m just abandoning them.”

Many shared the difficulty of being unable to care for their patients “on probably the worst day of their life.” Describing a recently arrived refugee patient, a participant said, “[She was] incredibly emotionally distraught in finding her diagnosis [of major fetal anomalies]. Just think, the psychological trauma that she was having to go through already. And then the idea of having to carry and continue this pregnancy to term….I just wanted to be able to do something or anything for her, and not being able to was really, really, really, really hard. And this was a noncompatible with life pregnancy—why couldn’t I help her?”

Those unable to provide care for unstable or high-risk patients at their own institutions described spending hours orchestrating hospital or inter-state transfer, cutting into time with other patients or their own families. Even after extensive time spent arranging handoffs, they feared poor outcomes. “How frustrating is it that somebody has to drive hours somewhere to get a medically recommended therapy, when the physician in the office [is] telling you, ‘I can take care of you but the law says I can’t’? Let’s say she had a bad outcome, let’s say she labored or started bleeding in the car…[how] frustrating it is that we’re limited in what we can and can’t do just to take care of people?”

### Personal Impacts

#### Moral Distress

Fifty of 54 participants (93%) reported situations in which they or their colleagues could not follow clinical standards due to legal constraints. When describing their moral distress over such encounters, participants used words like muzzled, handcuffed, and straitjacketed. One said, “That word moral injury is getting thrown around a lot now, but that’s what really this is…I know what the right thing is to do for my patients, but I am carrying this legal worry and worried about what could happen to my family at the same time. And that’s a terrible thing to feel.”

#### Perceived Consequences of Violating State Law

Most participants (47 [87%]) reported worries about practicing in an uncertain legal climate. Fears centered on potential for criminal prosecution, loss of medical license, loss of income, or incarceration. One discussed how these fears pervaded her everyday practice: “I feel like there’s a politician in the room with me with patients…just waiting to send me to jail, to make an example out of me if I say the wrong thing.” Another said, “it used to be that any day you were going to work, you could get sued. And now, any day you go to work, you could get sued or you could be charged with a felony. And that additional anxiety just weighs on me.” One participant recounted the visceral nature of this fear: “I’m in the [operating room] dry heaving. I’m not dry heaving because of this surgery. I know how to do this surgery. I trained for this surgery. I trained for the stress of treating an unstable…ectopic pregnancy. I did not train for, I am not ready for thinking about, ‘Is this the case that’s gonna make me a felon?’”

One participant described feeling torn between obligations to current and to future patients: “Bending the rules [for 1 patient] would compromise my ability to take care of the next 5000 people.” Others played out potential consequences of being charged, saying, “As soon as you’re charged with a felony, you might still have your medical license, but insurers aren’t gonna cover you…You’ll probably lose hospital privileges. It would basically be the end of your career.” Others also feared “being made publicly a villain.” A few retained private criminal defense attorneys at their own expense, fearing inadequate representation by their institution.

#### Leaving the State

Six participants (11%) had moved their practices to states with stronger abortion protections. When asked why, a participant said, “I moved to [former state] to serve that community because that’s who I care about…But I was having to send people across state lines for care that I could provide to them. And pitting my own livelihood against patient well-being, that was not a situation I was willing to be in anymore.” While 29 of 48 participants (60%) had entertained the idea of leaving their state, this was unfeasible for many, due to personal ties. For example, a participant said, “I am married to a man who has shared custody of 3 children…But I absolutely hate working here…I feel trapped here by my family situation.”

In contrast, some felt galvanized to stay. One explained, “I’ve thought so many times about leaving but I’m only 1 of 3 people, really, in this state who can take care of a patient who is possibly dying from their pregnancy. And that makes me want to stay.” Another said, “Restrictions anywhere open the door for restrictions everywhere, so it didn’t really make sense for me to leave at this point.”

As colleagues moved away, those who remained described shouldering increased burdens of clinical care and declining morale as colleagues moved away. Several mentioned challenges of filling vacant OB-GYN positions in abortion-restrictive states. One department chair said, “Hiring has been very hard….individuals that we have offered [an open] position to have ultimately decided either that they’re leaving [STATE] or that they’re not willing to have a job in [STATE]…And then it is retention. It’s trying to keep my [doctors] happy enough with the care that they’re able to give patients, that they don’t get fed up and leave.”

#### Mental Health

Most participants (38 [70%]) reported symptoms of anxiety and depression as a direct consequence of *Dobbs v Jackson*. One said, “When [*Roe*] was overturned, there was just a cloud that came over and it has not left.” Several reported not feeling fully present at home anymore: “There’s kind of a constant unease, a feeling of being just uneasy.” One participant, who sought counseling for these mental health impacts, said, “When I was in the Army, I deployed to Iraq…I left a 15-month-old baby at home with my husband. I practiced medicine in a war zone…but it’s never felt like this. My life was actually at risk in that scenario. I had to wear a flack vest and be armed when I was providing care…but I didn’t feel this way. I never had to see a counselor. I never had any treatment for mental health in all of those years.”

A minority of participants (16 [30%]) felt that their state’s ban had not increased stress levels, often because of abortion-restrictive institutional policies predating *Dobbs v Jackson.* Although men accounted for 19% of the overall sample (10 participants), they represented 38% of participants (6 of 16) reporting no mental health impacts.

## Discussion

This qualitative study identified complex, wide-ranging impacts of state abortion bans on OB-GYNs, leading to profound shifts in care provision and many questioning whether they could continue practicing in those states. This is 1 of the first empirical studies of *Dobbs v Jackson* as an occupational health hazard for OB-GYNs, in addition to a public health hazard for patients. We find that bans have, in many cases, placed heavy burdens on OB-GYNs by asking them to choose between standard patient care and their own legal exposure. Such cases leave lasting scars on OB-GYNs. In addition to widespread distress and anxiety, the state of hypervigilance we observed in several participants—constantly worrying about potential consequences of providing care or counseling—increases the risk for longer-term physical and mental health problems.^33^

These findings suggest the need for health care organizations to invest in efforts to support, attract, and retain OB-GYNs in restrictive states, to protect physicians’ own health and that of their patients. Some participants felt that institutions harmed OB-GYNs through overly conservative interpretation of laws, prioritizing institutional protection over ethical obligations to patients. Among OB-GYNs in such settings, we observed high levels of moral distress and turnover intention. In contrast, participants described a supportive institution as one that both had their back legally and also provided guidance for good clinical care in the current legal environment. Strong institutional support did not completely protect against adverse health and well-being impacts, but unsupportive institutions often amplified feelings of moral distress, poor mental health, and desire to leave.

Clinician burnout and mental health disorders are longstanding occupational health concerns for reproductive health care clinicians specifically^17,18^ and for physicians overall.^36^ Such concerns were brought to light and amplified by the COVID-19 pandemic.^37^ In the pandemic’s wake, trust in medicine is at an all-time low, particularly in politically conservative states.^38^ Our findings suggest that state abortion bans further threaten health, well-being, and professional integrity of OB-GYNs in these states.

### Limitations

This study has limitations. For example, nonprobabilistic sampling strategies carry the risk of selection bias. Those who chose to participate in interviews may have had stronger views on abortion laws than those who declined. However, studies find strong support of abortion rights among OB-GYNs.^34^ Compared with OB-GYNs nationally, study participants were more likely to self-identify as White (83% vs 61% nationally) and female (81% vs 62% nationally).^35^ In addition, this study was limited to OB-GYNs. Further research is needed on the impacts of *Dobbs v Jackson* on other reproductive health care clinicians (eg, family medicine physicians, nurse midwives). Study strengths include the participation of OB-GYNs from 13 affected states, the sample’s sociodemographic diversity, and launch of data collection within 9 months of the *Dobbs v Jackson* decision.

## Conclusions

The findings of this qualitative study suggest that state abortion bans have created an occupational health crisis for OB-GYNs intertwined with a maternal health crisis for their patients. These abortion bans may have implications for future availability of reproductive health care in much of the country. Given that states with the most restrictive laws were already among the states with highest rates of pregnancy-related mortality prior to the *Dobbs v Jackson* ruling,^39^ loss of clinicians in these states could further exacerbate geographic disparities in pregnancy outcomes. Supporting the OB-GYN workforce in the current policy environment is essential for preserving timely and accessible reproductive health care in the US.

## Notes

Supplement.
Data Sharing Statement
