# Supplementary material for: US Obstetrician-Gynecologists' Perceived Impacts of Post–Dobbs v Jackson State Abortion Bans
Source: JAMA Netw Open. 2024 Jan 17;7(1):e2352109. doi: 10.1001/jamanetworkopen.2023.52109 (PMC10794934; doi:10.1001/jamanetworkopen.2023.52109)
Supplement: Supplement. — Data Sharing Statement [file jamanetwopen-e2352109-s001.pdf]

Data Sharing Statement

Sabbath. Post–Dobbs v Jackson State Abortion Bans and US Obstetrician-Gynecologists' Perceived Impacts. *JAMA Netw Open*. Published January 17, 2024. doi:10.1001/jamanetworkopen.2023.52109

Data
Data available: No

Additional Information
Explanation for why data not available: This qualitative data is highly sensitive and, per our informed consent form, will never be made publicly accessible
